# Insomnia symptoms in women and men after percutaneous coronary intervention – a prospective multicentre cohort study

**DOI:** 10.1186/s41687-026-01122-0

**Published:** 2026-06-16

**Authors:** Aase Marie Aurbakken Rusten, Anders Broström, Tone M. Norekvål, Heather D. Hadjistavropoulos, Laila Haugsdal, Signe Stelling Risom, Tore Wentzel-Larsen, Trond Røed Pettersen

**Affiliations:** 1 Department of Medicine, Stord Hospital, Stord, Norway; 2 Department of Nursing, Jönköping University, Jönköping, Sweden; 3 Department of Clinical Neurophysiology, Linköping University Hospital, Linköping, Sweden; 4 Department of Heart Disease, Haukeland University Hospital, Bergen, Norway; 5 Faculty of Health and Social Sciences, Western Norway University of Applied Sciences, Bergen, Norway; 6 Department of Psychology, University of Regina, Regina, Canada; 7 Department of Cardiology, Herlev and Gentofte Hospital, Hellerup, Denmark; 8 Institute for Nursing and Nutrition, University College Copenhagen, Copenhagen, Denmark

**Keywords:** Coronary artery disease, Insomnia, Long-term follow-up, Self-reported health status, Sex differences, Sleep

## Abstract

**Aims:**

Insomnia is an independent risk factor for coronary artery disease (CAD). Persistent sleep difficulties after percutaneous coronary intervention (PCI) may hinder recovery, however, studies investigating factors contributing to insomnia in a longitudinal perspective are scarce. Therefore, we aimed to describe and determine: (i) the prevalence of insomnia symptoms in women and men with CAD after PCI; (ii) changes in insomnia symptoms scores over time and (iii) the association between insomnia symptoms, self-reported health status and individual factors 2-, 6- and 12 months post-PCI.

**Methods and results:**

Large-scale, prospective, multicentre cohort study on patients after PCI (*N* = 3,417). Patient-reported outcomes for all included patients were collected during index hospitalization after PCI prior to hospital discharge and 2-, 6-, and 12-months post-PCI (T0-T3 respectively). Insomnia symptoms were assessed with the Minimal Insomnia Symptom Scale, and self-reported health status with RAND-12 and the Seattle Angina Questionnaire. Clinical characteristics were collected from The Norwegian Registry of Invasive Cardiology and patients’ medical records. The prevalence of insomnia symptoms was high (women 47% vs. men 28%) at T0 (*p* < 0.001) and remained at similar levels (T1-T3). Reduced mental health in men (*p* < 0.001, T1, T2 and T3) and women (*p* = 0.002 (T1), *p* = 0.005 (T2) and *p* = 0.040 (T3)) and physical health in women (*p* = 0.032 (T2) and *p* = 0.016 (T3)) were significantly associated with more severe insomnia symptoms. Moreover, insomnia symptoms at previous timepoints were associated with insomnia symptoms at later timepoints for both sexes (*p* < 0.001 for all comparisons).

**Conclusion:**

Insomnia symptoms are highly prevalent following PCI, particularly among women, persisting over time, and related to poorer mental health in men and women. Greater attention should be directed toward early screening and targeted sleep interventions in both sexes.

**Graphical Abstract:**

**Figure:**
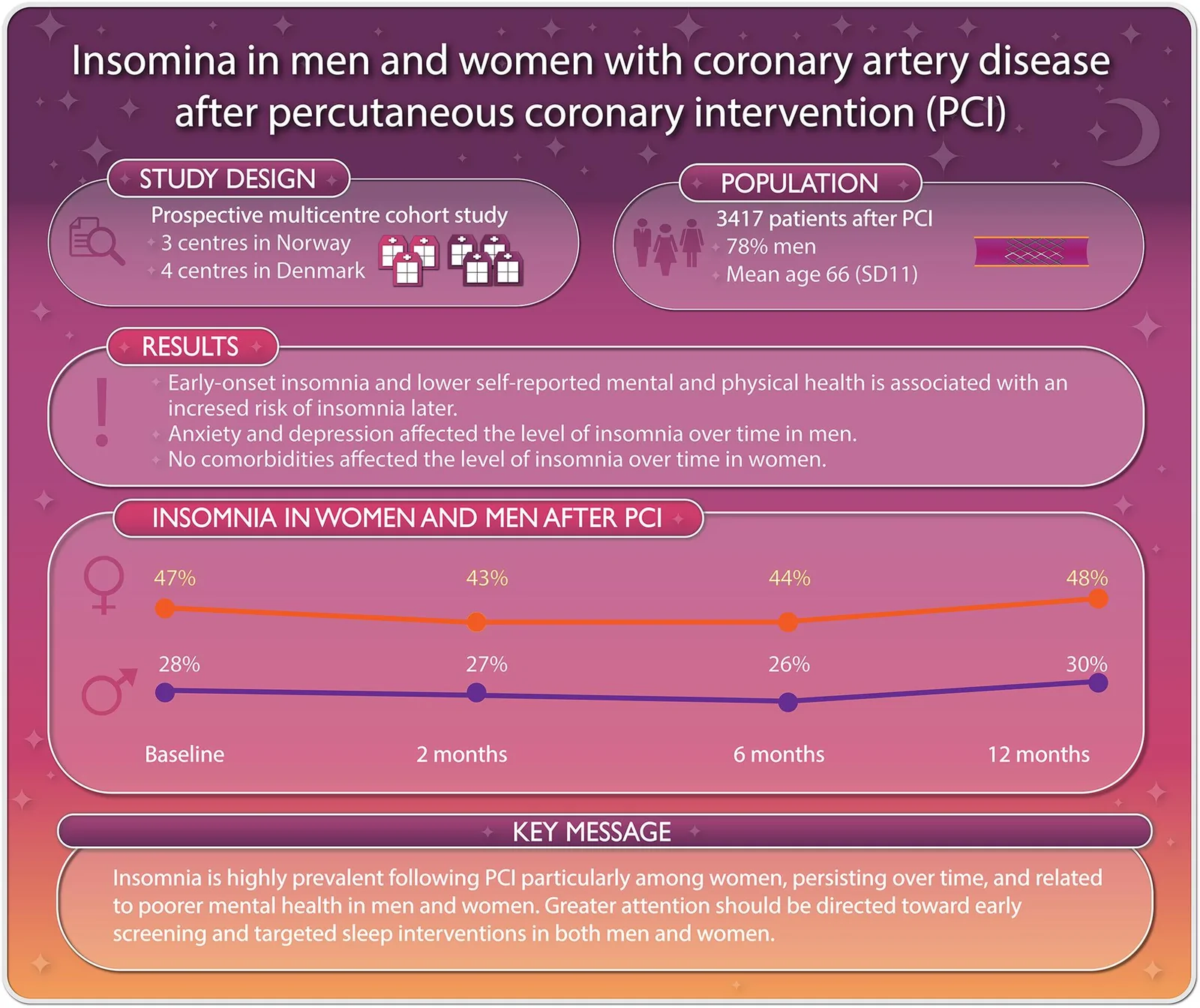

**Supplementary Information:**

The online version contains supplementary material available at 10.1186/s41687-026-01122-0.

## Introduction

In recent decades, there has been increasing awareness of the importance of sleep for good health [1], and insomnia represents an independent risk factor for both physical and mental health [2]. A strong association has been established between insomnia and coronary artery disease (CAD) [3, 4]. Insomnia may lead to disturbances in regulation of blood pressure, affect the nervous system, increase inflammation, and alter hormone secretion, thereby contributing to the development of atherosclerosis.[5] Both the European Society of Cardiology [6] and the American Heart Association [7] emphasize the importance of good and sufficient sleep to prevent CAD.

According to the World Health Organization’s International Classification of Diseases (ICD-11), the following diagnostic criteria must be met to be diagnosed with insomnia: Difficulties initiating sleep or maintaining sleep, leading to sleep dissatisfaction and reduced daytime functioning. If the symptoms last for more than three months the condition is referred to as chronic. The European Insomnia Guideline states that approximately 10% of adults in Europe suffers from chronic insomnia.[8] However, the prevalence of insomnia in patients with CAD is reported to be considerably higher – up to 45%[9–11].

Several factors threaten sleep quality in hospitalized patients.[12] Sleep disturbances are common during hospitalization following an acute cardiac event with subsequent percutaneous coronary intervention (PCI) before the sleep pattern gradually normalizes after hospital discharge.[13] However, previous studies had short follow-up periods, small sample sizes and substantial heterogeneity. Several factors contribute to the development of insomnia, including sex, with women having a substantially greater risk of developing insomnia than men.[5] Insomnia is also associated with significant daytime impairment, including fatigue, low mood or irritability, and reduced attention or concentration.[8] Furthermore, patients report excessive thinking and worrying at night, including concerns about their CAD, occupational stress, and fear of cardiac symptoms as possible causes of their sleep problem [14].

To understand how different factors may contribute to the development of insomnia, the 3P-Disease Model (3P-Model) provides a relevant theoretical framework to interpret and understand the findings in a wider context. This framework explains how various factors can predispose (e.g., sex, age, family history, psychopathology), precipitate (e.g., specific situations or events which trigger psychological stress) and perpetuate both insomnia and CAD (e.g., maladaptive behaviour and thoughts, chronic physical illness, coping style).[15] Despite substantial research on insomnia as a risk factor for developing CAD in both men and women,[3,16,17] research on insomnia in patients with established CAD is scarce. Increased knowledge about factors associated with insomnia following PCI is important to the clinical care of these patients, especially as insomnia may increase the risk of new cardiac events.[18] Therefore, we aimed to describe and determine: (i) the prevalence of insomnia symptoms in women and men with CAD after PCI; (ii) changes in insomnia symptoms scores over time and (iii) the association between insomnia symptoms, self-reported health status and individual factors 2-, 6- and 12 months post-PCI.

## Methods

### Study design and setting

This study draws data from the large-scale, prospective multicentre cohort study CONCARD^PCI^.[19] CONCARD^PCI^ was conducted at three PCI centres in Norway and four in Denmark between June 2017 and May 2020. Patient-reported outcomes were collected at four measuring points: baseline (T0), and follow-up at 2- (T1), 6- (T2), and 12 months (T3) after PCI.

### Study population

**Fig. 1:**
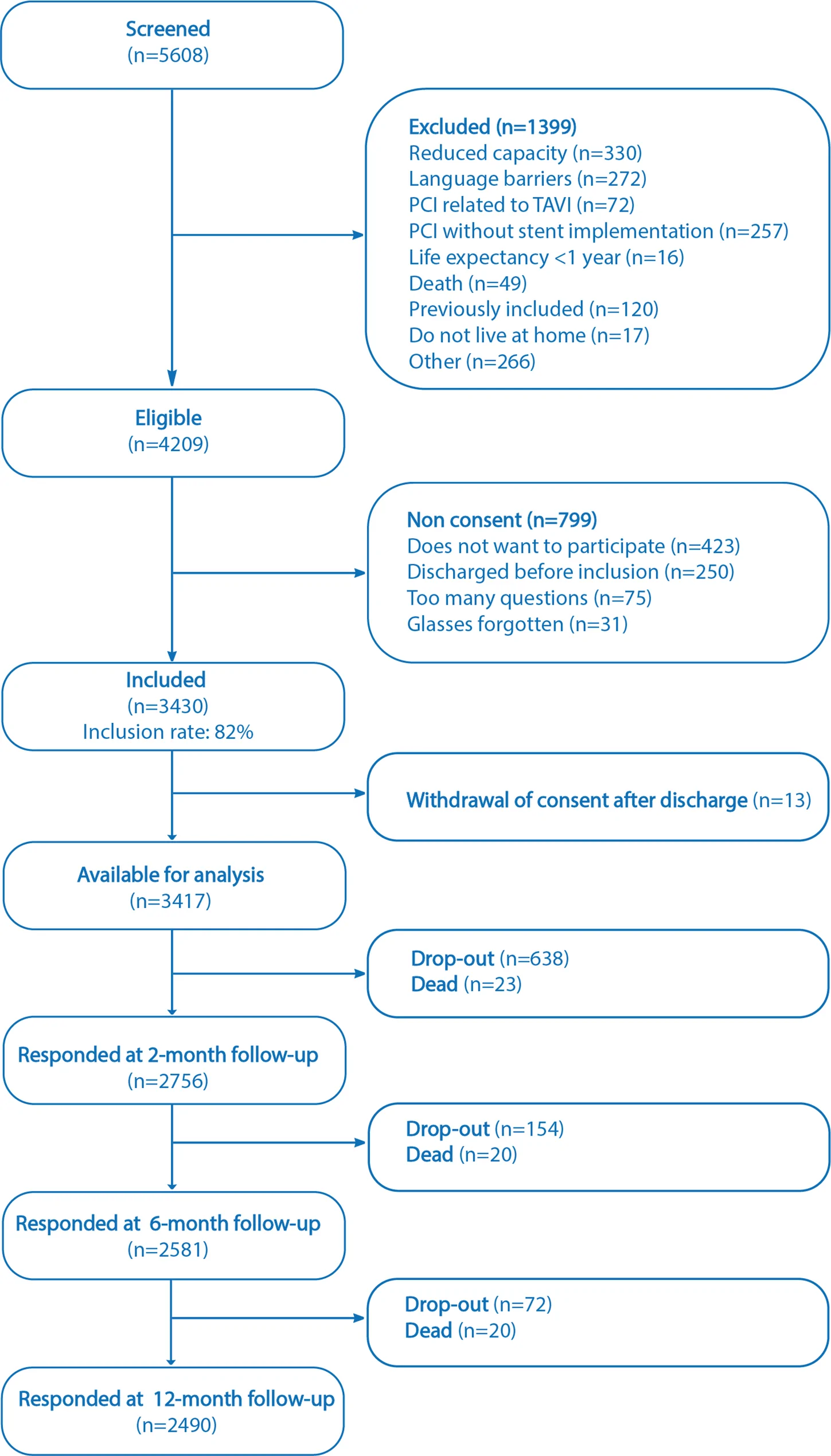
Patient flow in the study

The study population comprised all patients included in CONCARD^PCI^. To identify eligible patients, medical records and operating programs were reviewed. Trained CONCARD^PCI^ study nurses screened 5608 patients for eligibility during index hospitalization according to the following inclusion criteria: undergoing PCI, ≥ 18 years of age, community-dwelling and providing informed consent.[19] Of these, 1399 patients were excluded according to the following exclusion criteria: inability to speak Norwegian/Danish, impaired physical or mental capacity, in need of a proxy to complete the questionnaire, life expectancy < 1 year, undergoing PCI without stent implantation, PCI related to transcatheter aortic valve implantation or a MitraClip examination and previously enrolment in CONCARD^PCI^ (readmissions within the 12-month study period). If cognitive impairment was suspected and had not been previously reported, the 4-Attitude Test [20] and the Confusion Assessment Method [21] were used to screen the patients. Delirious or clinically unstable patients were reassessed for inclusion before hospital discharge. In total, 3430 patients consented to participate (inclusion rate 82%). Thirteen patients withdrew the consent after hospital discharge, leaving 3417 patients available for analysis at baseline (Fig. 1).

## Measures

### The minimal insomnia symptom scale (MISS)

MISS assesses the main features of insomnia symptoms: 1) difficulties initiating sleep, 2) difficulties maintaining sleep and 3) problems with early morning awakening.[22] A Likert scale ranging from ‘None’ (0), to ‘Very severe’ (4) provides a total sum score from 0 to 12. MISS has shown adequate reliability and validity and is a brief, easy-to-administer screening instrument. [22] For this study, the internal consistency of the scale was satisfactory at all measuring points (α = 0.76, α = 0.78, α = 0.75, and α = 0.76 at T0-T3 respectively). Although it is primarily intended as a screening rather than a diagnostic tool, a score of ≥ 6 points may characterize individuals with probable clinical insomnia. [22] Based on this, we dichotomized the MISS score into high and low insomnia symptom score groups using a cut-off of ≥ 6 points to estimate prevalence for Table 1 and the prevalence analyses.

**Table 1:** Baseline characteristics of patients undergoing percutaneous coronary intervention

Characteristics	Total study sample *N* = 3417* n (%)	High insomnia symptom score (MISS ≥6p) *n* = 990 (32%) *n* (%)	Low insomnia symptom score (MISS ≤6p) *n* = 2129 (68%) *n* (%)	*p*-value**	Adjusted *p*-value***
**Age**, mean (SD)	66 (11)	64 (11)	66 (11)	< 0.001	< 0.001
**BMI**, mean (SD)	27.7 (4.5)	27.9 (4.6)	27.7 (4.4)	0.259	1.000
**Sex**				< 0.001	< 0.001
Men Women	2671 (78) 746 (22)	681 (28) 309 (47)	1778 (72) 351 (53)		
**Living alone**	750 (24)	300 (42)	420 (58)	< 0.001	< 0.001
**Not living alone**	2340 (76)	641 (28)	1623 (72)		
**Ethnicity**				0.783	1.000
Native born Born of immigrant parents Immigrant	2824 (92) 114 (4) 135(4)	863 (32) 32 (31) 44 (34)	1872 (68) 72 (69) 84 (66)		
**Education**				< 0.001	< 0.001
Primary school Vocational school Upper secondary school University college or university < 4 years University college or university ≥ 4 years	639 (20) 1374 (43) 298 (9) 485 (15) 380 (12)	253 (42) 404 (30) 89 (31) 124 (26) 103 (27)	349 (58) 930 (70) 197 (69) 348 (74) 272 (73)		
**Employment**				< 0.001	< 0.001
Full-time work Part-time work Retired Other ****	1056 (33) 162 (5) 1615 (50) 366 (11)	277 (27) 61 (39) 456 (29) 185 (53)	754 (73) 95 (61) 1095 (71) 162 (47)		
**Indication for PCI**				< 0.001	0.008
Stable coronary disease Unstable angina pectoris NSTEMI STEMI Other	1023 (30) 441 (13) 916 (27) 742 (22) 294 (9)	338 (36) 146 (37) 231 (28) 197 (29) 78 (29)	608 (64) 252 (63) 592 (72) 486 (71) 190 (71)		
**Previous PCI**	875 (26)	297 (37)	511 (63)	< 0.001	< 0.001
**Previous CABG**	312 (9)	102 (36)	179 (64)	0.090	0.989
**Previous cardiovascular comorbidity** Atrial fibrillation/flutter Chronic heart failure Coronary artery disease Hypercholesterolemia Hypertension Myocardial infarction Peripheral artery disease	408 (12) 272 (8) 1163 (34) 1570 (46) 1774 (52) 704(21) 204 (6)	120 (33) 84 (34) 379 (36) 469 (33) 560 (35) 229 (36) 76 (42)	245 (67) 161 (66) 687 (64) 969 (67) 1035 (65) 416 (64) 105 (58)	0.626 0.371 0.001 0.399 < 0.001 0.022 0.002	1.000 1.000 0.016 1.000 0.001 0.269 0.033
**Previous medical comorbidity** Anxiety and depression Cancer Cerebrovascular disease Chronic obstructive pulmonary disease Chronic renal failure Diabetes (insulin) Diabetes (tablets)	333 (10) 395 (12) 214 (6) 247 (7) 157 (5) 210 (6) 495 (15)	144 (49) 124 (35) 65 (36) 90 (41) 46 (33) 87 (45) 156 (35)	149 (51) 229 (65) 117 (64) 132 (59) 93 (69) 106 (55) 289 (65)	< 0.001 0.155 0.248 0.004 0.740 < 0.001 0.102	< 0.001 < 0.001 1.000 0.047 1.000 0.001 1.000
**Smoking**	529 (17)	156 (32)	335 (68)	0.939	1.000
**Number of comorbidities**				< 0.001	< 0.001
No comorbidity One comorbidity Two comorbidities Three or more comorbidities	366 (11) 471 (14) 500 (15) 2080 (61)	81 (23) 110 (25) 122 (27) 677 (36)	265 (77) 330 (75) 337 (73) 1197 (64)		

Abbreviations: BMI: Body Mass Index; CABG: Coronary artery bypass grafting; NSTEMI: Non-ST-segment elevation myocardial infarction; STEMI: ST-segment elevation myocardial infarction; PCI: Percutaneous coronary intervention

*Discrepancy within the numbers because the total study sample (*N* = 3417) includes those with missing items on The Minimal Insomnia Symptom Scale (*n* = 3119) and other questionnaires. **P-value calculated using independent samples t-test or chi-square test. ***Holm correction for multiple comparisons. ****Other included those on sick leave, disability pension, seeking employment, students/compulsory military service, unpaid leave, or recipients of government support for various reasons

### The seattle angina questionnaire-7 (SAQ-7)

SAQ-7 is a seven-item disease-specific instrument assessing health status in patients with CAD. [23] This short version of SAQ comprises 3 domains measuring physical limitations, frequency of chest pain and quality of life. Responses are scored on a 5- and 6 item Likert scale and the scores are then converted to a 0-100 scale for each domain. A higher score indicates no limitations, no angina, or good quality of life. The SAQ-7 has shown satisfactory psychometric properties in patients with CAD undergoing PCI. For this study, the internal consistency of the physical limitations scale was satisfactory at T1 (α = 0.76) and T2 (α = 0.82). Internal consistency for the frequency of chest pain scale was low, which can be attributed to the small number of items (α = 0.60 at T1 and α = 0.65 at T2). However, the internal consistency of the quality-of-life scale was unacceptable and excluded from the analyses (α = 0.43 at T1 and α = 0.44 at T2).

### RAND-12

RAND-12 is a generic 12-item scale commonly used to assess self-reported health status. [24] It estimates disease burden and evaluates disease-specific benchmarks compared with other populations. Additionally, the scale encompasses eight domains of physical and mental health, which are summarized into two component scores: physical component summary (PCS) and mental component summary (MCS). Higher scores indicate better health-related quality of life. The instrument has shown satisfactory psychometric properties. [25].

### Data collection

A detailed description of the data collection can be found elsewhere. [19] In brief, sociodemographic data were self-reported at T0. Clinical characteristics were collected from The Norwegian Registry of Invasive Cardiology (NORIC) and patients’ medical records in Norway. Only medical records were used in Denmark. Norwegian patients received postal questionnaires while the Danish patients also had an electronic option (SurveyXact V.12.9). National registries were used to check vital status to avoid sending questionnaires to deceased patients or their relatives. Non-responders received one reminder.

### Statistical analysis

Descriptive statistics were used to present the data with mean, standard deviation and range for the continuous variables and absolute numbers and percentages for the categorical variables. T-tests were performed to examine the difference between patients with and without insomnia symptoms and continuous variables, and Pearson’s chi-square test for nominal variables. Holm correction was applied for multiple comparisons. Multiple linear regression analysis was performed to investigate the associations between self-reported health status and clinical- and sociodemographic factors, with insomnia symptoms as the dependent variable. The analyses were conducted separately for women and men. In Model 1, all independent variables were entered into the regression model simultaneously. In Model 2, a hierarchical approach was used, where insomnia symptoms at previous measuring points were added to the model. When computing the total score of the MISS, missing values were handled using the *half rule* technique. [26] The score was the average of the answered questions, provided that at least half of the questions have been answered. For the regression analyses, missing data were handled using multiple imputation. [27 Several imputed datasets were created, and the results of the analyses performed were subsequently pooled. In this study, we used 200 imputations, as in theory, using a higher number, is always better — especially if there is a high degree of uncertainty in the data. [28] Statistical significance was set at a p-value < 0.05. Analyses were performed in SPSS version 29 (IBM SPSS Statistics for Windows, Version 29.0. Armonk, NY: IBM Corp). Multiple regression analyses with multiple imputation were performed using the *mice* package in R statistical software version 4.4.1.

### Ethical considerations

This study conforms to the Helsinki Declaration (2008) and Norwegian and Danish legislation. Written informed consent was obtained from all participants, and the patients were informed that they could withdraw at any time and with no reason. The data are kept confidential and are stored in secured files at the hospital’s research server. The study was approved by the Norwegian Regional Committee for Ethics in Medical Research in Western Norway (REK 2015/57) and the Data Protection Agency in the Zealand region (REG-145-2017) in Denmark. CONCARD^PCI^ is registered in ClinicalTrials.gov (NCT03810612 - Initial Release: 12/21/2018).

## Results

### Population characteristics

Most patients were men (78%), had a mean age of 66 years (SD 11, range 20–96 years), and underwent PCI due to acute coronary syndrome (62%). Hypertension was present in 52% of the patients, and 26% had a history of PCI. Patient characteristics are presented in Table 1 and sex differences in clinical- and sociodemographic characteristics in Supplemental Table 1.

### Self-reported insomnia symptoms

**Fig. 2:**
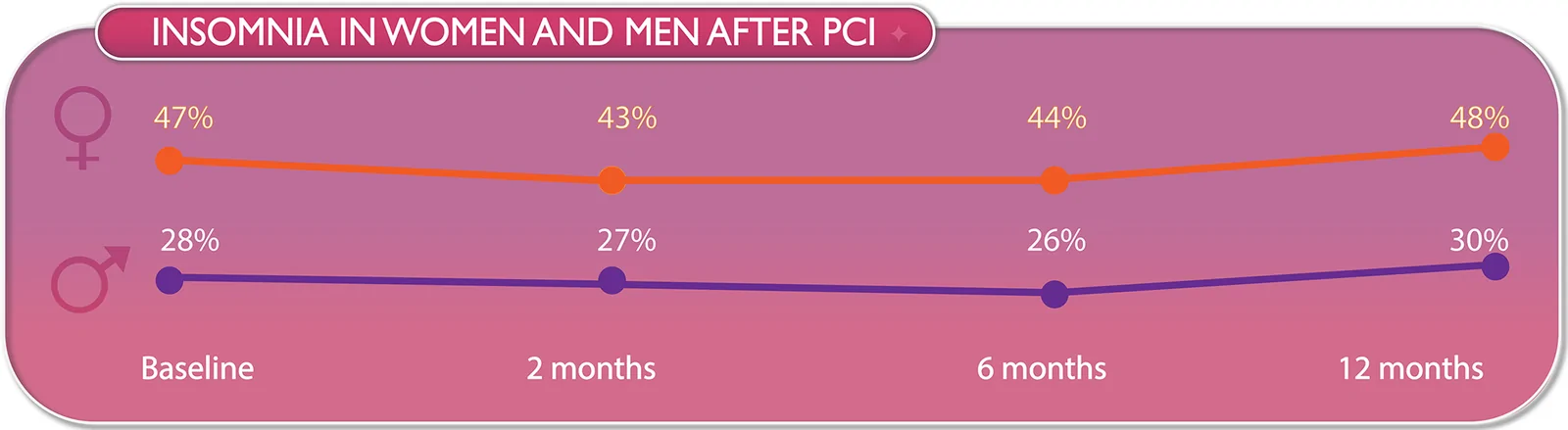
Prevalence of high insomnia symptom score by sex. Percentage women (red) and men (blue) with insomnia at different measurement points, (p<0.001 for all comparisons). Abbreviations: T0: Baseline; T1: 2-month follow-up; T2: 6-month follow-up; T3: 12-month follow-up

At T0, 32% of the total study sample reported insomnia symptoms. Patients with insomnia symptoms were slightly younger (64 vs. 66 years, *p* < 0.001), and more likely to live alone (42% vs. 28%, *p* < 0.001) compared to patients without insomnia symptoms. Furthermore, the prevalence of insomnia symptoms was higher in those with primary school as their highest level of education compared to those with vocational school, upper secondary school or university college or university education (*p* < 0.001). Insomnia symptoms were also significantly more prevalent in patients who were on sick leave, receiving disability benefits, were out of the workforce for other reasons and thus received government support, or who were working part-time, compared to those who worked full-time or were retired (*p* < 0.001). There was a significantly higher proportion of insomnia symptoms among patients with a history of prior PCI, hypertension, anxiety and depression and insulin treated diabetes (*p* < 0.001 for all comparisons) (Table 1). The prevalence of insomnia symptoms was significantly higher in women compared to men at all measuring points (*p* < 0.001 for all comparisons) (Fig. 2). For both women and men, the prevalence of insomnia symptoms showed a slight decline at T1 and T2, followed by an increase at T3. At T0, 37% of those with insomnia symptoms reported severe to very severe difficulties falling asleep in the evening. Interrupted sleep was a severe to very severe problem for 41%, and 43% reported severe to very severe problems with waking up too early in the morning. These difficulties remained relatively unchanged throughout the year following PCI. In the multiple linear regression analysis, younger age was significantly associated with higher levels of insomnia symptoms at all time points in both men and women (except for women at T2), but only in Model 1 (Tables 2, 3, 4, 5, 6, 7). Furthermore, in Model 1, higher level of insomnia symptoms was significantly associated with treated anxiety and depression in men at T1 (*p* = 0.015), T2 (*p* = 0.001) and T3 (*p* = 0.001) (Tables 3, 5 and 7), hypertension at T2 (*p* = 0.018) and previous PCI in women at T3 (*p* = 0.024) (Table 6). A significant association between polypharmacy (≥ 5 medications) and higher level of insomnia symptoms in men at T3 was observed in both Model 1 and Model 2 (*p* = 0.021 and < 0.001) (Table 7). In addition, living alone was associated with higher levels of insomnia in both sexes at different time points; in men at T1 (*p* = 0.001 (Model 1) and *p* = 0.043 (Model 2)) and at T2 (*p* < 0.001 (Model 1) and in women at T1 (*p* = 0.009 (Model 1) and *p* = 0.017 (Model 2)). Model 2 showed that higher level of insomnia symptoms at T0 was significantly associated with higher level of insomnia symptoms at T1 (*p* < 0.001), higher level of insomnia symptoms at T1 was significantly associated with higher level of insomnia symptoms at T2 (*p* < 0.001) and that higher level of insomnia symptoms at T2 was significantly associated with higher level of insomnia symptoms at T3 in both men and women (*p* < 0.001) (Tables 2, 3, 4, 5, 6 and 7).

**Table 2:** Multiple linear regression, insomnia symptoms among women at T1. **Model 1 and 2**

Variables	Coefficient model 1	Coefficient model 2	CI low model 1	CI low model 2	CI high model 1	CI high model 2	*p*-value model 1	*p*-value model 2
**Sociodemographic variables**								
Age (per 10 years)	-0.28	-0.15	-0.51	-0.34	-0.05	0.03	0.019	0.107
Live alone	0.64	0.45	0.16	0.08	1.12	0.81	0.009	0.017
Level of education							0.392	0.244
Vocational school	0.19	0.29	-0.37	-0.15	0.74	0.73	0.506	0.195
Upper secondary school	0.20	0.03	-0.53	-0.53	0.93	0.60	0.589	0.914
College/Uni. < 4 years	0.29	0.08	-0.45	-0.49	1.02	0.66	0.443	0.777
College/Uni./4 years or more	0.89	0.72	0.02	0.04	1.75	1.4	0.044	0.038
**Clinical variables**								
BMI	0.01	0.02	-0.04	-0.02	0.06	0.05	0.643	0.397
Smoking	0.55	0.27	-0.06	-0.22	1.15	0.76	0.078	0.274
Treated anxiety/depression	0.22	0.06	-0.51	-0.53	0.95	0.66	0.558	0.830
Hypertension	-0.03	-0.22	-0.53	-0.62	0.47	0.17	0.902	0.271
Insulin treated DM	0.31	-0.23	-0.50	-0.87	1.12	0.42	0.451	0.490
Previous PCI	0.60	0.19	-0.08	-0.35	1.28	0.73	0.086	0.490
Previous CABG	0.08	0.22	-0.92	-0.56	1.07	0.99	0.879	0.582
Polypharmacy (≥ 5 medications)	-0.42	-0.06	-1.00	-0.54	0.17	0.41	0.161	0.790
Number of years with CAD	0.01	0.01	-0.05	-0.03	0.06	0.05	0.821	0.530
**Self-reported physical and mental health**								
MCS12 T0*	-0.59	0.12	-0.97	-0.18	-0.22	0.42	0.002	0.435
PCS12 T0*	-0.34	-0.32	-0.79	-0.68	0.11	0.03	0.138	0.075
SAQ physical limitation T0*	-0.09	-0.03	-0.21	-0.12	0.03	0.07	0.143	0.582
SAQ angina frequency T0*	0.12	0.10	0.02	0.03	0.22	0.18	0.017	0.009
Insomnia symptoms sum score T0		0.68		0.61		0.74		< 0.001
R^2^ = 0.17 Adjusted R^2^ = 0.15 (model 1). R^2^ = 0.53 Adjusted R^2^ = 0.52 (model 2).								

Abbreviations: BMI: Body Mass Index; CABG: Coronary artery bypass grafting; CAD: Coronary artery disease; CI; Confidence Interval (95%); DM: diabetes mellitus; MCS Mental Composite Score; PCS: Physical Composite Score; PCI: Percutaneous coronary intervention; SAQ: The Seattle Angina Questionnaire. Level of education is all compared to primary school. T0 = During index hospitalization after PCI. *Per 10 points

**Table 3:** Multiple linear regression, insomnia symptoms among men at T1. **Model 1 and 2**

Variables	Coefficient model 1		Coefficient model 2	CI low model 1	CI low model 2	CI high model 1	CI high model 2	*p*-value model 1	*p*-value model 2
**Sociodemographic variables**									
Age (per 10 years)	-0.26		-0.09	-0.37	-0.18	-0.16	0.00	< 0.001	0.039
Live alone	0.49		0.24	0.21	0.01	0.77	0.47	0.001	0.043
Level of education								0.274	0.821
Vocational school	-0.30		-0.05	-0.61	-0.31	0.01	0.20	0.058	0.670
Upper secondary school	-0.07		0.07	-0.53	-0.3	0.38	0.45	0.758	0.702
College/Uni. < 4 years	-0.20		-0.02	-0.57	-0.33	0.17	0.28	0.297	0.879
College/Uni./4 years or more	-0.05		0.1	-0.44	-0.22	0.33	0.41	0.783	0.554
**Clinical variables**									
BMI	0.00		0.00	-0.02	-0.02	0.03	0.02	0.915	0.692
Smoking	-0.03		0.00	-0.31	-0.23	0.25	0.24	0.837	0.977
Treated anxiety/depression	0.63		0.06	0.12	-0.35	1.13	0.48	0.015	0.762
Hypertension	0.13		0.02	-0.09	-0.16	0.36	0.20	0.241	0.827
Insulin treated DM	0.10		0.16	-0.40	-0.26	0.60	0.58	0.689	0.446
Previous PCI	0.14		0.17	-0.14	-0.07	0.43	0.40	0.324	0.161
Previous CABG	-0.07		-0.03	-0.49	-0.37	0.35	0.32	0.744	0.881
Polypharmacy (≥ 5 medications)	-0.24		-0.14	-0.49	-0.35	0.02	0.07	0.071	0.194
Number of years with CAD	0.01		0.00	-0.01	-0.01	0.03	0.02	0.562	0.625
**Self-reported physical and mental health**									
MCS12 T0*	-0.75		-0.14	-0.95	-0.30	-0.55	0.03	< 0.001	0.096
PCS12 T0*	-0.19		-0.09	-0.42	-0.28	0.04	0.10	0.102	0.339
SAQ physical limitation T0*	0.02		0.03	-0.04	-0.02	0.08	0.08	0.560	0.193
SAQ angina frequency T0*	0.02		0.03	-0.03	-0.01	0.07	0.07	0.381	0.159
Insomnia symptoms sum score T0			0.66		0.62		0.70		< 0.001
R^2^ = 0.17 Adjusted R^2^ = 0.17 (model 1). R^2^ = 0.49 Adjusted R^2^ = 0.49 (model 2).									

Abbreviations: BMI: Body Mass Index; CABG: Coronary artery bypass grafting; CAD: Coronary artery disease; CI; Confidence Interval (95%); DM: diabetes mellitus; MCS Mental Composite Score; PCS: Physical Composite Score; PCI: Percutaneous coronary intervention; SAQ: The Seattle Angina Questionnaire. Level of education is all compared to primary school. T0 = During index hospitalization after PCI. *Per 10 points

**Table 4:** Multiple linear regression, insomnia symptoms among women at T2. **Model 1 and 2**

Variables	Coefficient model 1	Coefficient model 2	CI low model 1	CI low model 2	CI high model 1	CI high model 2	*p*-value model 1	*p*-value model 2
**Sociodemographic variables**								
Age (per 10 years)	-0.19	0.00	-0.43	-0.18	0.06	0.19	0.132	0.984
Live alone	0.14	-0.23	-0.32	-0.57	0.60	0.11	0.547	0.189
Level of education							0.674	0.679
Vocational school	0.11	-0.08	-0.44	-0.50	0.66	0.33	0.699	0.694
Upper secondary school	-0.06	0.12	-0.78	-0.43	0.66	0.67	0.873	0.667
College/Uni. < 4 years	0.30	0.30	-0.43	-0.27	1.03	0.87	0.426	0.300
College/Uni./4 years or more	0.56	0.10	-0.29	-0.54	1.4	0.74	0.195	0.759
**Clinical variables**								
BMI	-0.02	-0.01	-0.06	-0.05	0.03	0.02	0.484	0.502
Smoking	0.16	0.04	-0.53	-0.47	0.85	0.54	0.649	0.880
Treated anxiety/depression	0.58	0.42	-0.15	-0.15	1.30	0.99	0.118	0.145
Hypertension	0.00	0.06	-0.47	-0.3	0.47	0.42	0.998	0.750
Insulin treated DM	0.26	0.10	-0.53	-0.52	1.06	0.71	0.515	0.753
Previous PCI	0.40	0.05	-0.28	-0.49	1.09	0.6	0.247	0.850
Previous CABG	-0.09	-0.25	-1.13	-1.04	0.95	0.53	0.860	0.527
Polypharmacy (≥ 5 medications)	-0.39	-0.17	-0.94	-0.59	0.16	0.24	0.162	0.412
Number of years with CAD	0.00	0.00	-0.05	-0.04	0.05	0.04	0.955	0.816
**Self-reported physical and mental health**								
MCS12 T1*	-0.60	0.10	-1.01	-0.21	-0.19	0.41	0.005	0.537
PCS12 T1*	-0.56	-0.42	-1.08	-0.8	-0.05	-0.03	0.032	0.033
SAQ physical limitation T1*	0.05	0.12	-0.12	0.00	0.21	0.25	0.591	0.050
SAQ angina frequency T1*	-0.04	-0.06	-0.17	-0.16	0.09	0.04	0.520	0.276
Insomnia symptoms sum score T1		0.71		0.64		0.78		< 0.001
R^2^ = 0.21 Adjusted R^2^ = 0.19 (model 1). R^2^ = 0.59 Adjusted R^2^ = 0.58 (model 2).								

Abbreviations: BMI: Body Mass Index; CABG: Coronary artery bypass grafting; CAD: Coronary artery disease; CI; Confidence Interval (95%); DM: diabetes mellitus; MCS Mental Composite Score; PCS: Physical Composite Score; PCI: Percutaneous coronary intervention; SAQ: The Seattle Angina Questionnaire. Level of education is all compared to primary school. T1 = Two months follow up. *Per 10 points

**Table 5:** Multiple linear regression, insomnia symptoms among men at T2. **Model 1 and 2**

Variables	Coefficient model 1	Coefficient model 2	CI low model 1	CI low model 2	CI high model 1	CI high model 2	*p*-value model 1	*p*-value model 2
**Sociodemographic variables**								
Age (per 10 years)	-0.25	-0.08	-0.35	-0.16	-0.14	0.00	< 0.001	0.040
Live alone	0.48	0.15	0.21	-0.05	0.75	0.35	< 0.001	0.155
Level of education							0.281	0.856
Vocational school	-0.27	-0.05	-0.57	-0.26	0.02	0.17	0.067	0.676
Upper secondary school	-0.29	-0.11	-0.73	-0.44	0.14	0.21	0.189	0.493
College/Uni. < 4 years	-0.22	-0.09	-0.58	-0.36	0.14	0.17	0.222	0.499
College/Uni./4 years or more	-0.03	0.04	-0.41	-0.24	0.34	0.31	0.862	0.778
**Clinical variables**								
BMI	0.01	0.01	-0.01	-0.01	0.04	0.03	0.305	0.303
Smoking	-0.07	0.08	-0.45	-0.21	0.32	0.37	0.736	0.589
Treated anxiety/depression	0.84	0.30	0.36	-0.07	1.32	0.67	0.001	0.116
Hypertension	0.26	0.10	0.04	-0.06	0.47	0.26	0.018	0.220
Insulin treated DM	0.04	0.00	-0.43	-0.36	0.51	0.36	0.857	0.997
Previous PCI	0.10	0.02	-0.17	-0.20	0.38	0.23	0.466	0.889
Previous CABG	-0.08	-0.07	-0.49	-0.38	0.33	0.23	0.705	0.639
Polypharmacy (≥ 5 medications)	-0.12	0.04	-0.38	-0.15	0.13	0.23	0.341	0.694
Number of years with CAD	0.01	0.00	-0.02	-0.02	0.03	0.01	0.612	0.918
**Self-reported physical and mental health**								
MCS12 T1*	-0.73	-0.09	-0.93	-0.24	-0.53	0.06	< 0.001	0.229
PCS12 T1*	-0.24	-0.12	-0.47	-0.29	-0.01	0.05	0.040	0.173
SAQ physical limitation T1*	-0.02	0.04	-0.10	-0.03	0.07	0.10	0.673	0.253
SAQ angina frequency T1*	-0.05	-0.02	-0.12	-0.07	0.02	0.03	0.135	0.490
Insomnia symptoms sum score T1		0.71		0.68		0.74		< 0.001
R^2^ = 0.21 Adjusted R^2^ = 0.20 (model 1). R^2^ = 0.60 Adjusted R^2^ = 0.60 (model 2).								

Abbreviations: BMI: Body Mass Index; CABG: Coronary artery bypass grafting; CAD: Coronary artery disease; CI; Confidence Interval (95%); DM: diabetes mellitus; MCS Mental Composite Score; PCS: Physical Composite Score; PCI: Percutaneous coronary intervention; SAQ: The Seattle Angina Questionnaire. Level of education is all compared to primary school. T1 = Two months follow up. *Per 10 points

**Table 6:** Multiple linear regression, insomnia symptoms among women at T3. **Model 1 and 2**

Variables	Coefficient model 1	Coefficient model 2	CI low model 1	CI low model 2	CI high model 1	CI high model 2	*p*-value model 1	*p*-value model 2
**Sociodemographic variables**								
Age (per 10 years)	-0.30	-0.19	-0.56	-0.40	-0.05	0.02	0.020	0.072
Live alone	0.05	0.03	-0.43	-0.36	0.54	0.42	0.828	0.893
Level of education							0.671	0.306
Vocational school	0.34	0.19	-0.22	-0.27	0.89	0.66	0.234	0.416
Upper secondary school	-0.11	-0.34	-0.86	-0.97	0.64	0.28	0.776	0.281
College/Uni. <4 years	0.09	-0.22	-0.64	-0.82	0.81	0.38	0.812	0.477
College/Uni./4 years or more	0.14	-0.31	-0.72	-1.04	1.00	0.42	0.750	0.399
**Clinical variables**								
BMI	-0.02	-0.02	-0.07	-0.06	0.03	0.02	0.363	0.298
Smoking	0.50	0.40	-0.16	-0.14	1.16	0.94	0.138	0.142
Treated anxiety/depression	0.14	-0.06	-0.63	-0.71	0.90	0.59	0.726	0.852
Hypertension	0.16	0.12	-0.32	-0.29	0.65	0.52	0.515	0.578
Insulin treated DM	0.51	0.19	-0.34	-0.55	1.37	0.92	0.239	0.615
Previous PCI	0.77	0.47	0.10	-0.10	1.44	1.04	0.024	0.105
Previous CAGB	0.12	-0.05	-0.89	-0.90	1.13	0.80	0.818	0.905
Polypharmacy (≥ 5 medications)	-0.02	0.32	-0.58	-0.14	0.54	0.78	0.943	0.176
Number of years with CAD	-0.03	-0.04	-0.08	-0.08	0.01	0.01	0.166	0.107
**Self-reported physical and mental health**								
MCS12 T2*	-0.43	0.14	-0.84	-0.22	-0.02	0.49	0.040	0.456
PCS12 T2*	-0.58	-0.47	-1.06	-0.87	-0.11	-0.07	0.016	0.023
SAQ physical limitation T2*	-0.09	-0.04	-0.23	-0.16	0.05	0.08	0.231	0.538
SAQ angina frequency T2*	-0.02	-0.03	-0.15	-0.14	0.12	0.08	0.810	0.559
Insomnia symptoms sum score T2		0.62		0.55		0.69		< 0.001
R^2^ = 0.23 Adjusted R^2^ = 0.21 (model 1). R^2^ = 0.53 Adjusted R^2^ = 0.51 (model 2).								

Abbreviations: BMI: Body Mass Index; CABG: Coronary artery bypass grafting; CAD: Coronary artery disease; CI; Confidence Interval (95%); DM: diabetes mellitus; MCS Mental Composite Score; PCS: Physical Composite Score; PCI: Percutaneous coronary intervention; SAQ: The Seattle Angina Questionnaire. Level of education is all compared to primary school. T2 = Six months follow up. *Per 10 points

**Table 7:** Multiple linear regression, insomnia symptoms among men at T3. **Model 1 and 2**

Variables	Coefficient model 1	Coefficient model 2	CI low model 1			CI low model 2	CI hich model 1	CI high model 2	*p*-value model 1	*P*-value model 2
**Sociodemographic variables**										
Age (per 10 years)	-0.20	-0.03	-0.31		-0.12		-0.08	0.06	0.001	0.502
Live alone	0.21	-0.08	-0.08		-0.31		0.50	0.15	0.154	0.482
Level of education									0.173	0.298
Vocational school	-0.38	-0.19	-0.69		-0.45		-0.06	0.06	0.020	0.133
Upper secondary school	-0.38	-0.25	-0.83		-0.61		0.08	0.11	0.102	0.172
College/Uni. < 4 years	-0.29	-0.14	-0.67		-0.44		0.09	0.17	0.135	0.375
College/Uni./4 years or more	-0.35	-0.33	-0.75		-0.64		0.04	-0.02	0.080	0.036
**Clinical variables**										
BMI	0.01	0.00	-0.014		-0.02		0.03	0.02	0.428	1.000
Smoking	0.19	0.23	-0.22		-0.08		0.59	0.55	0.362	0.148
Treated anxiety/depression	0.88	0.42	0.36		-0.01		1.40	0.86	0.001	0.056
Hypertension	0.20	-0.02	-0.02		-0.19		0.43	0.16	0.076	0.864
Insulin treated DM	0.13	0.17	-0.38		-0.24		0.64	0.59	0.613	0.406
Previous PCI	-0.01	-0.06	-0.31		-0.3		0.30	0.18	0.971	0.608
Previous CABG	-0.40	-0.23	-0.83		-0.56		0.03	0.11	0.067	0.187
Polypharmacy (≥ 5 medications)	0.32	0.45	0.05		0.23		0.59	0.67	0.021	< 0.001
Number of years with CAD	0.01	0.01	-0.01		-0.01		0.03	0.02	0.461	0.553
**Self-reported physical and mental health**										
MCS12 T2*	-0.78	-0.06	-1.01	-0.25			-0.55	0.13	< 0.001	0.533
PCS12 T2*	-0.18	-0.08	-0.44	-0.29			0.09	0.12	0.192	0.423
SAQ physical limitation T2*	0.05	0.03	-0.03	-0.03			0.13	0.09	0.247	0.358
SAQ angina frequency T2*	-0.16	-0.08	-0.24	-0.14			-0.08	-0.02	< 0.001	0.010
Insomnia symptoms sum score T2		0.72		0.68				0.76		< 0.001
R^2^ = 0.20 Adjusted R^2^ = 0.19 (model 1). R^2^ = 0.57 Adjusted R^2^ = 0.56 (model 2).										

Abbreviations: BMI: Body Mass Index; CABG: Coronary artery bypass grafting; CAD: Coronary artery disease; CI; Confidence Interval (95%); DM: diabetes mellitus; MCS Mental Composite Score; PCS: Physical Composite Score; PCI: Percutaneous coronary intervention; SAQ: The Seattle Angina Questionnaire. Level of education is all compared to primary school. T2 = Six months follow up. *Per 10 points

### Self-reported mental and physical health (MCS and PCS)

Lower MCS at T0 was significantly associated with a higher level of insomnia symptoms at T1 in women (*p* = 0.002) and men (*p* < 0.001). Lower MCS at T1 and T2 were also significantly associated with a higher level of insomnia symptoms at T2 and T3 in men (*p* < 0.001) (Model 1, Table 5), but not when insomnia symptoms at previous measuring points were included (Model 2, Table 5). For women, both lower MCS and PCS were significantly associated with a higher level of insomnia symptoms in Model 1. Lower MCS at T1 was significantly associated with a higher level of insomnia symptoms at T2 (*p* = 0.005) and lower MCS at T2 was significantly associated with a higher level of insomnia symptoms at T3 (*p* = 0.040). In addition, lower PCS at T1 and T2 was significantly associated with a higher level of insomnia symptoms at T2 and T3 (*p* = 0.032 and *p* = 0.016). In Model 2, lower PCS at T1 remained significantly associated with a higher level of insomnia symptoms at T2 (*p* = 0.033) (Table 4) and lower PCS at T2 remained significantly associated with a higher level of insomnia symptoms at T3 (*p* = 0.023) (Table 6).

### Physical limitation and angina frequency

Higher angina frequency at T0 was significantly associated with increased insomnia symptoms at T1 among women (*p* = 0.017 in Model 1; *p* = 0.009 in Model 2) (Table 2). Among men, higher angina frequency at T2 was significantly associated with increased insomnia symptoms at T3 (*p* < 0.001 in Model 1), and this association remained significant in Model 2 (*p* = 0.010) (Table 7). Additionally, we found that among women, greater physical limitations at T1 were significantly associated with a higher level of insomnia symptoms at T2 (*p* = 0.050), but only in Model 2 (Table 4).

## Discussion

In this prospective multicentre cohort study of patients after PCI, a substantial proportion of both women and men, reported insomnia symptoms. Approximately half of the women reported insomnia symptoms, which was significantly higher than among men – and this remained stable over time. Lower self-reported health and a higher level of insomnia symptoms at a given time during the follow-up period were significantly associated with a higher level of insomnia symptoms at later measuring points. Patients with insomnia symptoms were slightly younger and this finding was significant in both men and women. However, there were significant sex differences in the clinical- and sociodemographic variables associated with level of insomnia symptoms over time, with lower self-reported mental and physical health and a history of PCI in women, and lower self-reported mental health in men. Living alone was associated with higher levels of insomnia symptoms in both sexes at different time points. Anxiety and depression and hypertension significantly affected the level of insomnia symptoms negatively over time in men, while no comorbidities significantly affected the level of insomnia symptoms negatively over time in women. In the following, findings of the study are discussed in accordance with the theoretical framework of the 3P-Model. Primarily, the 3P model is applied to explain psychological and behavioural mechanisms of insomnia. However, for this study, the model was employed as an organizational and interpretive model. According to the 3P-Model, various factors can predispose (e.g., sex, age, family history, psychopathology), precipitate (e.g., specific situations or events which trigger psychological stress) and perpetuate both insomnia symptoms and CAD (e.g., maladaptive behaviour and thoughts, chronic physical illness, coping style).[15].

### Predisposing factors

In our study, insomnia symptoms were significantly associated with younger age, similar to findings in other studies on patients with CAD. [9,11] Conversely, insomnia is associated with older age in the general population. [8,29] The proportion of women with insomnia symptoms was substantial in our study, which is consistent with findings from other studies reporting a higher risk of insomnia among women than men. [8,14,30] Up to 50% of women in the general population report sleep problems in midlife, [30] and underlying causes may involve changes associated with the menopausal transition, including fluctuations in hormone levels and vasomotor symptoms. [31] Moreover, family studies indicate that insomnia is moderately heritable, more strongly so in women compared to men. [32] Given that insomnia is associated with CAD, special attention should be given to sleep in women with CAD to reduce the risk of recurrent cardiac events. Known risk factors for CAD, such as anxiety and depression, are also associated with insomnia in the CAD population, [10,11] as observed in our study. Nearly half of the population that reported insomnia symptoms had a history of anxiety and depression. The regression analysis showed that being treated for anxiety and depression was significantly associated with a higher level of insomnia symptoms, however, only in men. Further, lower self-reported mental health before discharge and early in the remission period was significantly associated with the development of insomnia symptoms later. Psychological factors can have a predisposing and perpetuating function, in both insomnia and CAD. [15] Thus, patients’ mental health should be considered in preventive and therapeutic interventions to prevent both conditions. [33].

### Precipitating factors

In our study, all patients experienced symptoms of CAD followed by medical intervention and hospitalization - factors that may serve as a precipitating cause of acute insomnia. Like other studies,[13] the prevalence of insomnia symptoms was high around the time of PCI. However, it was anticipated that sleep patterns would normalize in the months following PCI, [13] and to some extent, this was observed. A high level of perceived distress during an acute coronary event, including greater fear of dying and feelings of helplessness, was associated with acute insomnia symptoms in another study. [10] Therefore, clinicians should pay close attention to how the patient is feeling during the hospital stay to identify any fear or distress.

### Perpetuating factors

We found that lower self-reported mental- and physical health at two- and six months were significantly associated with a higher level of insomnia symptoms at subsequent measuring points, suggesting that these are perpetuating factors likely contribute to the development of chronic insomnia. Chronic physical illness may be a factor that contributes to the persistence of sleep difficulties. [15] In addition, previous studies have found that diabetes and liver disease are significantly associated with insomnia in patients with CAD. [9,11] In the general population, insomnia has been linked to chronic conditions such as neurological disorders, diabetes, hypertension, cancer, and chronic obstructive pulmonary disease. [8] These conditions may also serve as predisposing factors. [15] Based on our findings it is warranted to consider implementing screening for insomnia symptoms for all patients, regardless of comorbidities. Insomnia symptoms were significantly more common among men living alone, compared to cohabiting men, as well as among women early in the study period. Lallukka et al. also reported higher rates of insomnia among individuals living alone in the general population. [29] Living alone may contribute to a perceived lack of social support among patients with insomnia and CAD, which is a key element in the recovery phase. [15] Thus, clinicians should maintain heightened awareness of this patient population. In some cases, individuals may adopt behaviours to compensate for or cope with the event that triggered acute insomnia, which in turn can lead to negative thoughts about sleep. This highlights the role of behaviour, cognition, and emotions across the levels of the 3P-Model and may contribute to the perpetuation of insomnia. [15] Clinicians should consider this and encourage patients to adopt to healthy coping strategies regarding CAD.

We found that more frequent episodes of angina at previous measuring points were significantly associated with higher levels of insomnia symptoms in women at early measuring point and in men at later measuring point. This indicate that angina symptoms increase the risk of developing chronic insomnia and calls for a greater focus on symptom relief, as well as thorough assessment of the underlying causes of these symptoms. Cardiac rehabilitation can improve health-related quality of life and may reduce symptoms of depression and anxiety in addition to increased physical activity. [6] According to the 3P-Model, improved health-related quality of life, reduced depression, and increased physical activity may contribute to prevention of the maintenance of insomnia, as well as stabilizing CAD. In our study, women in particular reported reduced PCS and physical limitations, and this was associated with higher insomnia symptom scores. In general, women are less physical active than men and are less likely to engage in cardiac rehabilitation compared to men. [6] This is concerning as physical activity is not only recommended in preventing new cardiac events but could also serve as a coping strategy. Thus, we should ensure referral to and actively recommend both women and men to participate in cardiac rehabilitation.

### Strength and limitations

This large prospective multicentre cohort study presents real-world data from two countries, including a large study sample (*N* = 3417). We achieved a high inclusion rate (82%) and high response rates at the subsequent measuring points (81%, 76% and 73% at T1-T3 respectively). To ensure relevance of the research questions and the choice of instruments, two patient representatives with a history of CAD participated in the planning and implementation of the study. The questionnaires were discussed with the patient representatives and piloted at each measuring point before the study was conducted. [20] Furthermore, the questionnaire assessed insomnia symptoms from the past four weeks, providing insight into sleep quality prior to the current cardiac event.

However, this study is not without limitations. First, potential participants were excluded if they did not speak Norwegian or Danish, which may affect the generalizability of the results. Still, this only applied to a small proportion of patients (*n* = 272). Second, only 22% of the study population were women. However, the sex and age distribution in this study corresponds to data from national health registries for the same patient population,[34] strengthening the generalizability of the results. Norwegian patients who declined participation were older compared to those who participated and more often had other indications for PCI than those participating (Supplemental Table 2). Still, the likelihood of responding increased with age among those who remained in the study (Supplemental Table 3). Finally, as the study relies on self-reported data, there is a potential for recall bias.

## Conclusion

Insomnia symptoms are prevalent among patients with CAD, with women being the most affected, and the prevalence remains high one year after PCI. Lower self-reported health and higher level of insomnia symptoms were associated with a higher level of insomnia symptoms at later measuring points. Lower self-reported mental and physical health and a history of PCI were found to be associated with a higher level of insomnia symptoms in women, and lower self-reported mental health and living alone in men. Also, anxiety and depression and hypertension added to the level of insomnia symptoms over time in men.

This highlights the need for healthcare professionals to pay close attention to and support patients at higher risk of insomnia symptoms. Systematic screening in clinical practice could potentially identify these high-risk patients, allowing preventive measures to be planned.

## Supplementary Information

Below is the link to the electronic supplementary material.

Supplementary Material 1

## Data Availability

The datasets generated and/or analysed during the current study are not publicly available according to the Norwegian data protection legislation. Analysis files (SPSS syntaxes, R scripts etc.) will be available from the corresponding author upon reasonable request.
