# Supplementary material for: Insomnia symptoms in women and men after percutaneous coronary intervention – a prospective multicentre cohort study
Source: J Patient Rep Outcomes. 2026 Jun 16;10:155. doi: 10.1186/s41687-026-01122-0 (PMC13507006; doi:10.1186/s41687-026-01122-0)
Supplement: Supplementary file 1 [file 41687_2026_1122_MOESM1_ESM.docx]

**Supplemental Table 1**. Sex differences in clinical- and sociodemographic characteristics.

| **Characteristics** | Total study sample N=3417  n (%) | Men  n=2671 (78%)  n (%) | Women  n=746 (22%)  n (%) | p-value* | Adjusted  p-value** |
| --- | --- | --- | --- | --- | --- |
| **Age,** mean (SD) | 66 (11) | 65 (11) | 68 (10) | <0.001 | <0.001 |
| **BMI,** mean (SD) | 27.7 (4.5) | 27.5 (4.4) | 28.3 (4.7) | <0.001 | 0.001 |
| **Living alone**  **Not living alone** | 750 (24)  2340 (76) | 493 (20)  1932 (80) | 257 (39)  408 (61) | <0.001 | <0.001 |
| **Ethnicity**  Native born  Born of immigrant parents  Immigrant | 2824 (92)  114 (4)  135(4) | 2233 (92)  75 (3)  114 (5) | 591 (91)  39 (6)  21 (3) | 0.001 | 0.011 |
| **Education**  Primary school  Vocational school  Upper secondary school  University college or university < 4 years  University college or university ≥ 4 years | 639 (20)  1374 (43)  298 (9)  485 (15)  380 (12) | 425 (17)  1128 (45)  217 (9)  400 (16)  325 (13) | 214 (31)  246 (36)  81 (12)  85 (12)  55 (8) | <0.001 | <0.001 |
| **Employment**  Full-time work  Part-time work  Retired  Other **** | 1056 (33)  162 (5)  1615 (50)  366 (11) | 945 (38)  104 (4)  1202 (48)  262 (10) | 111 (16)  58 (8)  413 (60)  104 (15) | <0.001 | <0.001 |
| **Indication for PCI**  Stable coronary disease  Unstable angina pectoris  NSTEMI  STEMI  Other | 1023 (30)  441 (13)  916 (27)  742 (22)  294 (9) | 799 (30)  340 (13)  694 (26)  609 (23)  228 (9) | 224 (30)  101 (14)  222 (30)  133 (18)  66 (9) | 0.039 | 0.310 |
| **Previous PCI** | 875 (26) | 730 (27) | 145 (20) | <0.001 | <0.001 |
| **Previous CABG** | 312 (9) | 262 (10) | 50 (7) | 0.010 | 0.107 |
| **Previous cardiovascular comorbidity**  Atrial fibrillation/flutter  Chronic heart failure  Coronary artery disease  Hypercholesterolemia  Hypertension  Myocardial infarction  Peripheral artery disease | 408 (12)  272 (8)  1163 (34)  1570 (46)  1774 (52)  704(21)  204 (6) | 329 (12)  229 (9)  950 (36)  1196 (45)  1326 (50)  588 (22)  156 (6) | 79 (11)  43 (6)  213 (29)  374 (51)  448 (61)  116 (16)  48 (6) | 0.211  0.013  <0.001  0.008  <0.001  <0.001  0.550 | 1.000  0.126  0.006  0.099  <0.001  0.002  1.000 |
| **Previous medical comorbidity**  Anxiety and depression  Cancer  Cerebrovascular disease  Chronic obstructive pulmonary disease  Chronic renal failure  Diabetes (insulin)  Diabetes (tablets) | 333 (10)  395 (12)  214 (6)  247 (7)  157 (5)  210 (6)  495 (15) | 221 (8)  306 (12)  172 (6)  178 (7)  130 (5)  145 (5)  401 (15) | 112 (15)  89 (12)  42 (6)  69 (9)  27 (4)  65 (9)  94 (13) | <0.001  0.664  0.452  0.014  0.154  0.001  0.103 | <0.001  1.000  1.000  0.126  0.924  0.011  0.720 |
| **Smoking** | 529 (17) | 419 (17) | 110 (15) | 0.334 | 1.000 |
| **Number of comorbidities**  No comorbidity  One comorbidity  Two comorbidities  Three or more comorbidities | 366 (11)  471 (14)  500 (15)  2080 (61) | 322 (12)  378 (14)  396 (15)  1575 (59) | 44 (6)  93 (12)  104 (14)  505 (68) | <0.001 | <0.001 |

Abbreviations: BMI: Body Mass Index; CABG: Coronary artery bypass grafting; NSTEMI: Non-ST-segment elevation myocardial infarction; STEMI: ST-segment elevation myocardial infarction; PCI: Percutaneous coronary intervention.

*P-value calculated using independent samples t-test or chi-square test.

**Holm correction for multiple comparisons.

***Other included those on sick leave, disability pension, seeking employment, students/compulsory military service, unpaid leave, or recipients of government support for various reasons.

**Supplemental Table 2.** Comparison of participants and those declining participation in the study for the Norwegian centres.

Logistic regression was performed to investigate relationships of sex, age and indication for percutaneous coronary intervention with participation.

^a^Joint adjustment for sex, age and indication.

^b^Odds ratio per 5 years.

^c^Other include arrhythmia examination, completion of previous percutaneous coronary intervention, heart failure/cardiomyopathy, non-specific chest pain, risk assessment following successful thrombolysis, suspected complications following angiography/percutaneous coronary intervention, valvular heart disease.

Abbreviations: CI: confidence intervals, OR: odds ratio, ref: reference, SD: standard deviation.

|  | **Participants (n=1970)**  **n (%)** | **Non-participants (n=582)**  **n (%)** | **OR** | **95% CI** | **p-value** | **OR** | **95% CI** | **p-value** |
| --- | --- | --- | --- | --- | --- | --- | --- | --- |
|  | **Descriptives** | | **Unadjusted analysis** | | | **Adjusted analysis^a^** | | |
| **Sex**  Female vs male (ref) | 1543 (78) | 428 (73) | 0.77 | 0.62 – 0.95 | 0.016 | 0.85 | 0.68 – 1.06 | 0.151 |
| **Age** (mean, SD)^b^ | 66 (11) | 69 (12) | 0.87 | 0.83 – 0.90 | <0.001 | 0.87 | 0.83 – 0.91 | <0.001 |
| **Indication for percutaneous coronary intervention**  Stable coronary artery disease (ref)  Unstable angina pectoris  Non-ST-segment elevation myocardial infarction  ST-segment elevation myocardial infarction  Other^c^ | 535 (27)  306 (16)  578 (29)  406 (21)  145 (7) | 120 (21)  86 (15)  183 (31)  105 (18)  84 (14) | 0.80  0.71  0.87  0.39 | 0.59 – 1.09  0.55 – 0.92  0.65 – 1.16  0.28 – 0.54 | <0.001    0.155  0.009  0.339  <0.001 | 0.79  0.74  0.79  0.37 | 0.57 – 1.08  0.57 – 0.96  0.59 – 1.07  0.27 – 0.52 | <0.001  0.133  0.022  0.127  <0.001 |

**Supplemental Table 3.** Comparison of responders and non-responders at two-month follow-up.

Logistic regression was performed to investigate relationships of sex, age and indication for percutaneous coronary intervention with participation.

^a^Joint adjustment for sex, age and indication.

^b^Odds ratio per 5 years.

^c^Other include arrhythmia examination, completion of previous percutaneous coronary intervention, heart failure/cardiomyopathy, non-specific chest pain, risk assessment following successful thrombolysis, suspected complications following angiography/percutaneous coronary intervention, valvular heart disease.

Abbreviations: CI: confidence intervals, OR: odds ratio, ref: reference, SD: standard deviation.

|  | **Responders T1 (n=2675)**  **n (%)** | **Non-responders T1 (n=661)**  **n (%)** | **OR** | **95% CI** | **p-value** | **OR** | **95% CI** | **p-value** |
| --- | --- | --- | --- | --- | --- | --- | --- | --- |
|  | **Descriptives** | | **Unadjusted analysis Adjusted analysis^a^** | | | | | |
| **Sex**  Female vs male | 2094 (78) | 496 (75) | 0.97 | 0.80 – 1.18 | 0.763 | 0.90 | 0.73 – 1.09 | 0.276 |
| **Age** (mean, SD)^b^ | 66 (10) | 64 (12) | 1.12 | 1.08 – 1.17 | <0.001 | 1.12 | 1.08 – 1.17 | <0.001 |
| **Indication for percutaneous coronary intervention**  Stable coronary artery disease (ref)  Unstable angina pectoris  Non-ST-segment elevation myocardial infarction  ST-segment elevation myocardial infarction  Other^c^ | 825 (31)  335 (13)  721 (27)  575 (22)  219 (8) | 198 (27)  106 (14)  195 (26)  167 (23)  76 (10) | 0.76  0.89  0.83  0.69 | 0.58 – 0.99  0.71 – 1.11  0.66 – 1.04  0.51 – 0.94 | 0.094  0.043  0.291  0.107  0.017 | 0.80  0.90  0.89  0.70 | 0.61 – 1.05  0.72 – 1.12  0.70 – 1.12  0.52 – 0.95 | 0.182  0.104  0.331  0.318  0.022 |
